# Becoming Tibetan: from millet to barley cultivation

**DOI:** 10.1093/nsr/nwz111

**Published:** 2019-09-13

**Authors:** Hans-Jürgen Bandelt

**Affiliations:** Department of Mathematics, University of Hamburg, Germany

The settlement of humans on the Tibetan Plateau has been widely investigated in archaeology and other fields [1]. Recent archaeological evidence has indicated that permanent human occupation on the Tibetan Plateau generally began around 3.6 thousand years ago (kya) and was likely facilitated by the introduction of barley agriculture. However, it remained unknown how barley agriculture spread onto the Plateau. Millet farmers had settled in lower-altitude regions in the north-eastern Tibetan Plateau by 5.2 kya, at the peak of a warm period, and adopted barley cultivation by 4.0 kya, at a time when an abrupt climatic shift was recorded in the eastern margin of the Tibetan Plateau. The hypothesis was raised that it was the millet farmers in the lower altitudes who eventually brought barley agriculture to the higher elevations.

How might genetics contribute to this question? To support the hypothesis, Li *et al.* [2] employed the highest-resolution mitochondrial DNA (mtDNA) data, i.e. entire mitogenomes, of modern Tibetans and neighbouring population groups. Based on phylogeographic analyses, the authors were able to pinpoint mtDNA haplogroups M9a1a1c1b1a and A11a1a as having local variation on the Tibetan Plateau and a potential origin in northern China during the Yangshao period. Bayesian analysis further predicted that both haplogroups together were more than twice as frequent (49%) at 3.6 kya than in modern Tibetans (21%). The authors interpreted these matrilineal haplogroups as potential genetic relics of the Neolithic millet farmers and suggested that substantial genetic components of contemporary Tibetans can thus trace their ancestry directly to those early millet farmers.

There are some caveats though. First, the higher proportions of the highlighted haplogroups in ‘Tibetans’ at 3.6 kya likely signify that (while barley was already traded) the potential newcomers were not yet part of a common Tibetan population and that the actual influx took place more gradually. It has been stressed that ‘caution should be exercised when discussing when and where agriculture was carried out on the Tibetan Plateau and with what permanence it was occupied’ [3] and, one may add, when discussing who exactly were the main actors in each region over time and how contact operated through trade networks. From the (matrilineal) genetic evidence, one cannot draw a picture of how the actual interaction between different groups of people on the Tibetan Plateau (and its margins) facilitated the adoption of various agricultural practices. What we can see through the lens of genetics is a kind of summary of gene flow at different times between groups of people that had their ancestries in geographically quite distant regions.

Li *et al.* have successfully demonstrated with mtDNA analysis that the Tibetan Plateau had eventually received an influx of descendants of people from the Yangshao period of northern China. But the entire intermediate phase of regional contacts, possibly via Sichuan [4,5], is difficult to reconstruct with genetic data alone. A full understanding of the prehistory of an area needs complex models to be worked out in close cooperation with several disciplines.
